# Smart Contact Lenses—A Step towards Non-Invasive Continuous Eye Health Monitoring

**DOI:** 10.3390/bios13100933

**Published:** 2023-10-18

**Authors:** Nikolay L. Kazanskiy, Svetlana N. Khonina, Muhammad A. Butt

**Affiliations:** 1 Samara National Research University, 443086 Samara, Russia; 2 IPSI RAS-Branch of the FSRC “Crystallography and Photonics” RAS, 443001 Samara, Russia

**Keywords:** smart contact lenses, non-invasive, continuous health monitoring, intraocular pressure, glucose monitoring

## Abstract

According to the age-old adage, while eyes are often considered the gateway to the soul, they might also provide insights into a more pragmatic aspect of our health: blood sugar levels. This potential breakthrough could be realized through the development of smart contact lenses (SCLs). Although contact lenses were first developed for eyesight correction, new uses have recently become available. In the near future, it might be possible to monitor a variety of ocular and systemic disorders using contact lens sensors. Within the realm of glaucoma, SCLs present a novel prospect, offering a potentially superior avenue compared to traditional management techniques. These lenses introduce the possibility of non-invasive and continuous monitoring of intraocular pressure (IOP) while also enabling the personalized administration of medication as and when needed. This convergence holds great promise for advancing glaucoma care. In this review, recent developments in SCLs, including their potential applications, such as IOP and glucose monitoring, are briefly discussed.

## 1. Introduction

Today, there exists a wide array of diagnostic tools capable of assessing a myriad of analytes that concern medical practitioners [1,2]. Regrettably, most of these tools are not designed to be worn and still predominantly rely on traditional blood draws and conventional bench-top assay methods. Consequently, a pivotal inquiry into the minds of many revolves around the following: How can wearable sensor technology transition towards monitoring more specific physiological phenomena? [3,4]. For example, this could involve confirming the well-being of a fetus by tracking mechanical motion within the mother’s womb, distinguishing a potentially hazardous seizure event from mere physical exertion, notifying athletes or laborers about impending dangerous dehydration, informing health-conscious individuals about the impact of highly-refined white bread on their blood glucose levels, or even facilitating the early detection and containment of viral infections within a population, well in advance of the manifestation of symptoms in the majority of individuals.

Wearable healthcare monitoring devices have been introduced because of recent developments in the major fields of electronics, materials, and biology and their integration with the Internet of Things (IoT) [5,6,7,8,9,10]. These devices can non-invasively sample bodily fluids like blood, interstitial fluid, sweat, urine, saliva, and tears and transmit the measured data to smartphones and healthcare departments, who then use it to provide periodic information about the patients’ conditions that is related to diagnostic, monitoring, and therapeutics [11,12]. These devices also help to decentralize healthcare, relieve pressure on healthcare sectors, and provide highly personalized and real-time healthcare data at the point of need [13]. It is predicted that in the next 25 years, the global healthcare sector will save USD 200 billion [14].

Within the realm of wearable healthcare devices, significant commercial interest has been directed towards SCLs due to their potential for healthcare applications [15,16]. The cornea’s surface offers a distinct and effortless interface to monitor physiological states within the human body. With direct connections to the brain, liver, heart, lungs, and kidneys, the eyes can act as a valuable gateway to observing the body’s conditions [17]. The tear has been transformed into a practical biological interface as a wearable medical device for out-of-hospital and self-monitoring applications since it is continuously and non-invasively accessible [18,19]. The development of SCLs, which can continuously sample tear fluid, assessing physiological conditions, and wirelessly transmit data to an electronic device like a smartphone that can then send the information to the appropriate healthcare facilities, is the result of recent advancements in integrated circuits (ICs) and biosensors coupled with wireless data communication techniques [20]. One important feature of wearable biosensors is continuous analyte monitoring. Despite having various advantages over other on-skin wearable medical devices, SCLs cannot include batteries for continuous power supply due to space constraints [11,21].

During the early 1970s, the Food and Drug Administration (FDA) approved the first commercial soft contact lenses, marking a significant milestone in the field [22]. Subsequently, there was a continual drive for improvement in soft contact lens technology, with a particular focus on enhancing their oxygen permeability and water absorption capabilities. It was not until the late 1990s and the early 2000s that silicon hydrogel emerged as the preferred material among contact lens manufacturers [23]. Silicon hydrogel lenses boasted an impressive fivefold increase in oxygen permeability, revolutionizing the industry by promoting prolonged eye hydration, health, and comfort compared to earlier options.

Innovative wearables known as SCLs are created to be worn on the cornea of the eye, just like conventional contact lenses. However, what makes them unique is how they incorporate cutting-edge technology to offer a variety of capabilities beyond simple vision correction [24]. These elements may include data visualization, augmented reality (AR) capabilities, and health monitoring, among others. SCLs are made from biocompatible materials to ensure they are safe for extended wear on the eye’s surface. They must be comfortable and not cause irritation or harm to the eye. Many of the cutting-edge contact lenses currently available have limitations, such as the ability to monitor only one parameter at a time or the need for multiple materials to achieve multifunctionality. In a study [16], the development of a flexible multifunctional contact lens utilizing inorganic γ-Fe_2_O_3_@NiO magnetic oxide nanosheets is proposed. This innovative lens seamlessly conforms to the eyeball, enabling the simultaneous monitoring of glucose levels in tears, eyeball movement, and intraocular pressure (IOP). The optimized contact lens design boasts impressive features, including a dependable glucose detection limit of 0.43 μmol, exceptional accuracy in measuring eye movement (95.27%), and a high sensitivity to IOP (0.17 MHz mmHg^− 1^). This groundbreaking research introduces a novel approach to integrated biochemical and biophysical sensing of ocular signals through contact lenses, facilitated by our innovative material. It offers a personalized and efficient solution for health management [16].

SCLs may have sensors that can keep an eye on several physiological factors. These sensors may assess things like IOP, glucose levels in tears, and even certain biomarkers for particular diseases [25,26]. The contact lenses contain minuscule microelectronics and processing units that process data from the sensors, carry out calculations, and connect to other devices. These glasses’ electronics need a power source to function. Microbatteries or energy-harvesting devices that transform incoming energy (such as light) into electrical power can be used to do this [27]. SCLs have the potential to incorporate displays that superimpose information onto what the wearer sees. This could involve displaying notifications, providing navigation instructions, and showing details. These advanced contact lenses can establish connections with devices, like smartphones, tablets, or computers. This allows for data transfer, real-time communication, and even the remote control of functionalities [27].

AR-enabled SCLs can superimpose digital images and information onto the wearer’s natural vision [28]. This allows for interactive and immersive experiences, such as navigation cues, gaming elements, and real-time data display [29,30]. SCLs can help people monitor their health in addition to correcting their eyesight by giving them real-time data on vital signs, biomarkers, and other health-related information [31]. Early medical condition detection may benefit from this. Although the idea behind SCLs is intriguing, it should be noted that this technology is still in its infancy. Before these devices can be extensively used, issues including miniaturization, power management, safety, and regulatory permissions need to be resolved. The limits of what is feasible with wearable technology are nonetheless being pushed by continual study and innovation in this area.

The paper is organized in the following manner. Section 2 provides a brief overview of the market size of SCLs. Section 3 is devoted to the materials, electrical components, fabrication methods, and applications of SCLs. We mainly focused on two major applications of SCLs that are being widely researched in current times. The paper ends with concluding remarks presented in Section 4. 

## 2. Market Size of SCLs

**Figure 1:**
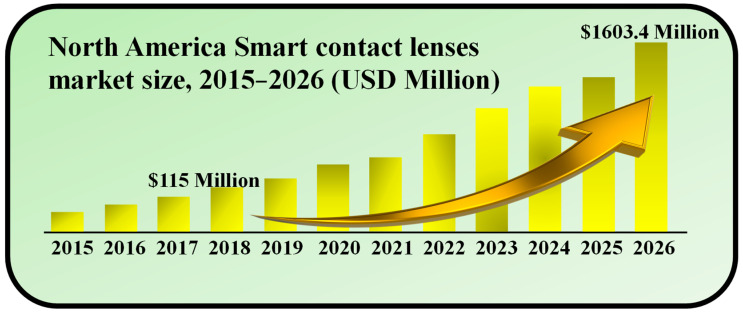
Market size of SCLs during 2015–2026. Inspired by [32].

In 2018, the size of the worldwide market for SCLs was valued at USD 115.0 million. Projections indicate that this market is anticipated to achieve a value of USD 1603.4 million by 2026, demonstrating a remarkable compound annual growth rate (CAGR) of 38.9% over the forecast period [32]. SCLs represent a recent leap in wearable electronics, offering the remarkable ability to monitor ocular and tear fluid physiological data. They offer immediate, non-intrusive medical insights into conditions like diabetes and glaucoma. Looking ahead, the potential of SCL technology includes photography through ocular capture and adaptive responses to ambient light changes. These lenses are poised to aid individuals with age-related macular degeneration (AMD) and address a multitude of other applications [33]. The escalating prevalence of diabetes and glaucoma plays a pivotal role in propelling the growth of the SCLs market during the forecast period. Furthermore, increased investment in research for advanced wearable electronics is anticipated to steer the market’s trajectory in the years to come, as shown in Figure 1.

## 3. Materials and Applications of SCLs

SCLs have found applications in the continuous monitoring of ocular parameters, encompassing both physical characteristics, such as pressure and temperature, as well as chemical markers like glucose levels, protein content, and pH [34,35]. These physiological indicators are intricately linked to human well-being, with noteworthy implications for health. For instance, elevated IOP can precipitate glaucoma, abnormal ocular surface temperatures can give rise to dry eye syndrome, and elevated tear glucose levels may serve as an early indicator of diabetic retinopathy [36,37,38].

**Figure 2:**
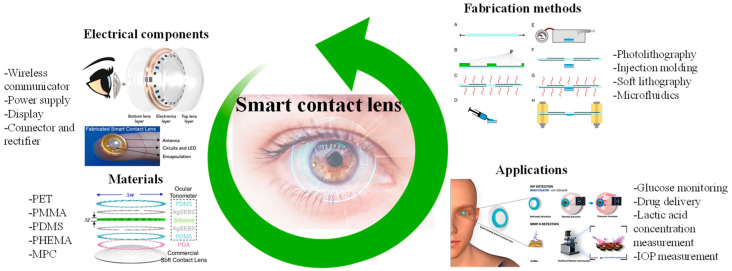
Graphical illustration depicting a summary of materials, electrical components, fabrication methods, and the potential applications of SCLs. Inspired by [44,45,46,47,48,49].

The non-invasive tracking of human health facilitated by SCLs promises a deeper comprehension of ocular and systemic physiological conditions. Consequently, it enables the timely implementation of effective measures for the early prevention or treatment of specific ailments. By enabling real-time monitoring of personal health data, SCLs eliminate the need for frequent hospital visits or reliance on bulky medical equipment [39,40,41]. Consequently, the development of SCL devices, particularly considering the rapid advancements in Internet of Things (IoT) technology, emerges as a pivotal research area [42,43]. In this section, the materials suitable for SCLs, electrical components used to activate the lenses, fabrication methods, and applications are briefly explained and shown in Figure 2. We mainly focused on two widely explored applications of SCLs, i.e., IOP and glucose monitoring. 

### 3.1. Materials

Modern contact lenses can be made from a variety of materials, including silicone [50], 2-methacryloyloxyethyl phosphorylcholine (MPC) [51], poly (methyl methacrylate) [52], poly (ethylene terephthalate) [51], poly (2-hydroxyethyl methacrylate) [53], and poly (ethylene terephthalate) [54]. For biomedical applications, SCLs made of silicone or poly(2-hydroxyethyl methacrylate) PHEMA hydrogels are preferred [55]. Designing and manufacturing SCLs for IOP monitoring presents a range of intricate challenges [56]. The specific needs of the human eye necessitate careful consideration of factors, such as flexibility, the clarity of the viewing window, hydrophilicity, and oxygen permeability [57,58]. Notably, pHEMA hydrogel—a prevalent material in commercial contact lenses—offers advantageous traits, like water permeability and compatibility with ocular tissue. This ensures the prolonged safe and comfortable usage of SCLs. However, a notable obstacle arises in the fusion of hydrogel-based lenses with electronic circuits, particularly rigid chips, and bulky batteries. This hurdle is attributable to the alterations in structure caused by the hydrogel’s tendency to swell. Ulu et al. created an antibacterial contact lens for the treatment of ocular infections using PHEMA hydrogel that was loaded with the antibacterial chemical boric acid [59]. A hydrogel based on PHEMA was created by Ashtiani et al. as a therapeutic contact lens for the administration of small-molecule medications [60]. Chitosan was used to alter the hydrogel in their investigation. Additionally, the therapeutic contact lens demonstrated antimicrobial efficacy and consistent ascorbic acid release. Most widely used contact lens materials are presented in Table 1.

To minimize deposition-related expenses, researchers have delved into the utilization of graphene. Graphene is renowned for its outstanding electrical, chemical, and mechanical attributes, as well as its optical transparency. Furthermore, the tightly knit hexagonal lattice structure of graphene effectively inhibits water infiltration, suggesting its potential application in preventing eye dehydration. Additionally, graphene exhibits exceptional electromagnetic wave shielding capabilities, with partial absorption of electromagnetic waves when incorporated into contact lenses [66]. A monolayer of graphene, previously cultivated on copper foil, is transferred onto the contact lens using a solution-based method and subsequently patterned using lithography [67]. However, it is worth noting that the lithography procedure can lead to increased manufacturing costs. To address this concern, Tang et al. employed more cost-effective techniques, specifically drop-casting and direct laser interference printing (DLIP), to deposit single-layer graphene onto contact lenses. In drop-casting, a droplet of graphene solution is placed on the contact lens and then vaporized. DLIP, on the other hand, involves positioning the graphene-coated lens between two mirrors, where a laser beam induces interference patterns on the graphene film, resulting in the removal of graphene from areas with high-laser intensity [67].

The wireless SCLs, utilizing a flexible inductor–capacitor–resistor (LCR) sensor devoid of chips and batteries, holds immense promise for monitoring physiological signals. To facilitate the adoption of LCR contact lenses for clinical intraocular pressure monitoring, it is imperative to employ reliable and comfortable contact lens materials while achieving exceptional sensitivity. A novel approach to creating hydrogel-based SCLs for wireless IOP monitoring involves the conformal stacking technique, effectively addressing hydrogel swelling and seamless integration of pyramid-micro-structured dielectric elastomers [37]. The system successfully monitors IOP in an in vitro porcine eye, thanks to the high sensitivity of the spherical pyramid-micro-structured capacitive pressure sensor and the hydrogel substrate. Furthermore, an impedance-matching tunable reader integrated into glasses enhances signal amplitude and extends the reading distance, thus improving the portability of signal measurement equipment. These innovations signify the substantial potential of the wireless contact lens system for clinical IOP monitoring, heralding a promising future for advanced daily ocular health management [37].

### 3.2. Electrical Components

The ideal design criteria for SCLs should allow for simple measurement and real-time display of ocular parameters without interfering with the patient’s routine activities. As a result, this kind of technology needs to be adaptable, small, and capable of integrating with a variety of functional modules, such as wireless communicators, sensors, powers, displays, and other micro-components [68].

The best technique to transmit signals in wearable electronics is through a wireless system as opposed to more conventional cable data transmission methods. To avoid the hassle of cable transmission, Chiou et al. created a wireless SCL system made up of a wireless communicator [69]. For real-time biomarker monitoring, SCLs require a reliable, long-lasting power source. However, due to their diminutive size, contact lenses have a limited capability for storing energy. Consequently, external sources of power (such as inductive power, radio frequency [RF] power, or optical power) must be used to wirelessly power biosensors. Radio waves are used by RFID, a type of identification technology, to identify individuals or things. Power harvesting from radio waves became possible with the development of passive RFIDs [70]. A strategy for recovering energy from the outside environment is power harvesting. This method enables the replacement of tiny batteries in low-power electrical appliances.

In the past decade, the pursuit of next-generation electronics, spurred by breakthroughs in materials science, has catalyzed the emergence of stretchable and transparent electronics [71,72]. This technological evolution has paved the way for innovative applications like SCLs and wearable sensors, which demand a more extensive investigation into the materials and manufacturing methods involved. Consequently, numerous research endeavors have been directed towards creating materials and devices that possess both mechanical stretchability and optical transparency. Utilizing flexible and biologically stable electrode materials is important for smart lenses to show pertinent information [73]. In this context, Lee et al. demonstrated a straightforward microscale light-emitting diode (LED) device made of graphene, a type of 2D carbon nanomaterial with superior optical, electrical, and mechanical capabilities, and constructed on a contact lens [66]. Additionally, graphene exhibits good air permeability and electromagnetic wave absorption, making it a suitable candidate for electromagnetic interference shielding. Another sort of wireless display was created by Park et al. [31]. It has three electronic parts: a rectifier, an antenna, and an LED pixel. These components were created on a Si wafer that had an 800 nm-thick Cu sacrificial layer placed on top of it. An inductively coupled alternating current (AC) was consequently wirelessly received by the contact lens from a transmission coil (50 MHz) within a 5 mm range.

Developing wearable devices capable of wirelessly monitoring IOP and facilitating precise medication administration is a pressing need in glaucoma treatment. However, this endeavor poses considerable challenges related to size constraints, wireless functionality, and interference issues. To address these challenges, an integrated wireless theragnostic contact lens is proposed for the real-time electrical monitoring of IOP and on-demand delivery of anti-glaucoma drugs [74]. This groundbreaking wireless theragnostic contact lens adopts an exceptionally compact structural design, allowing for seamless integration and efficient frequency separation on the curved and limited surface area of the lens. The IOP sensing component exhibits remarkable sensitivity, owing to its innovative cantilever configuration within the capacitive sensing circuit. Meanwhile, the drug delivery mechanism utilizes an efficient wireless power transfer circuit to trigger the release of anti-glaucoma medication into the aqueous chamber through iontophoresis. With its minimally invasive, intelligent, wireless, and theragnostic capabilities, the wireless theragnostic contact lens emerges as a highly promising solution for advancing glaucoma treatments [74].

Connecting the electrical components within SCLs requires microwires. For printing 2D or 3D flexible electronics, these wires are commonly constructed out of conductive silver or carbon nanotube inks [75]. These are used frequently because they are more affordable than gold and platinum. Silver ink is very adaptable for a wide range of applications due to its strong electrical and thermal properties. In recent years, a variety of conductive silver inks have been created for use as conductors for flexible paper displays, silver art inks with luminous LEDs, and miniature antennas that can be 3D-printed. Similar to this, printing techniques for numerous electronic components, such as emitters, radiofrequency inductors, and transistors, have extensively utilized carbon nanotubes with excellent mechanical and electrical qualities. Integrating wireless communication, power systems, displays, and various other elements, intelligent contact lenses facilitate effortless and non-intrusive physiological monitoring. These advancements surpass traditional methods dependent on inflexible circuit boards, cables, needle electrodes, and terminal connections.

**Figure 3:**
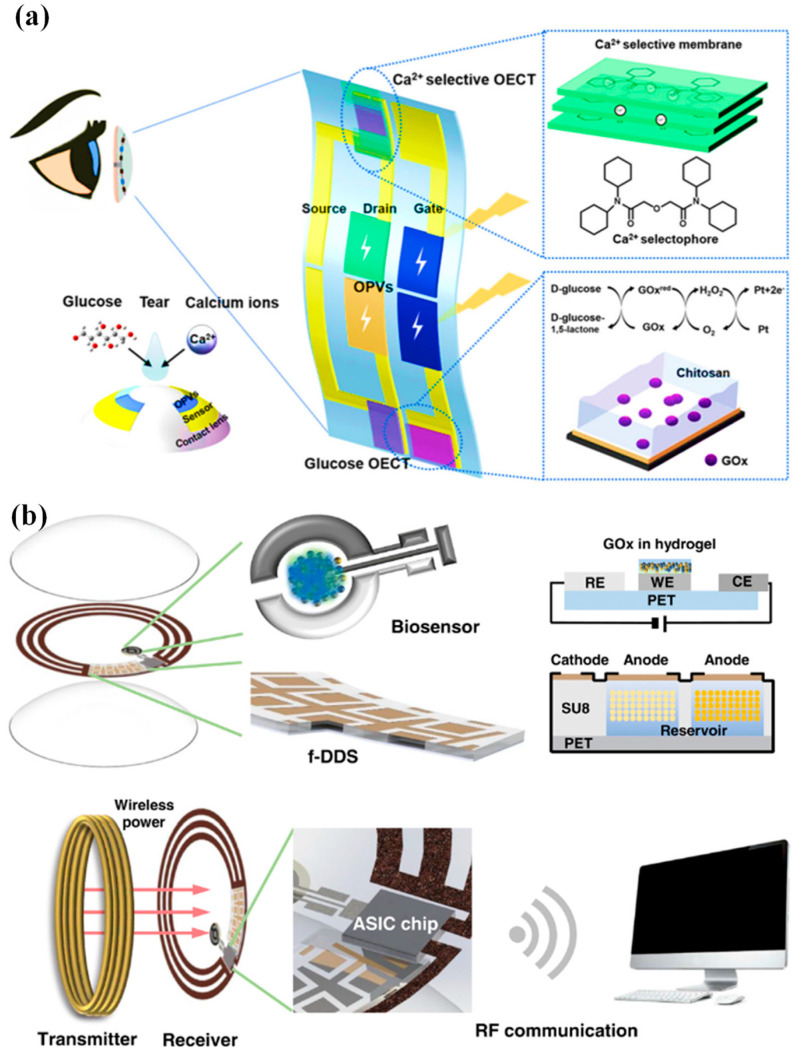
(**a**) Device and contact lens integration schematic. The electrode for the Ca^2+^ and glucose sensors has been modified, as shown in the insets [76]. (**b**) A visual representation of the innovative SCL engineered for both diabetic diagnosis and therapy. This advanced contact lens seamlessly integrates multiple key components, including a cutting-edge biosensor, a flexible on-demand drug delivery system (f-DDS), a wireless power transmission system with transmitter and receiver coils, an application-specific integrated circuit (ASIC) chip, and a versatile remote communication system. Together, these components form a versatile and ubiquitous platform with the potential to cater to a wide range of diagnostic and therapeutic applications, marking a significant advancement in the field of healthcare technology [24].

Moreover, multiplexed organic electrochemical transistor-based sensors are shown to be self-powered by organic solar cells (OSCs) [76]. The integrated device was created using a straightforward technique that included heat evaporation and solution blade coating. Without any peripheral circuitry, OSCs were tuned to produce the best operating voltage for sensors that respond semi-log-linearly to the calcium and glucose ions in tear fluids. A near-field communication unit can wirelessly communicate the sensing signals to the laptop. Real-time monitoring of the biomarkers in tears will be provided by an integrated self-powered multiplexed sensing device, which is anticipated to be put on SCLs for the early identification and diagnosis of diabetes. The design of the integrated system of multiplexed sensing devices for glucose and Ca^2+^ monitoring driven by OSCs is shown in Figure 3a. The two components of this system are (i) two groups of OSCs with one inverted cell powering the gate electrode, and (ii) two separate PEDOT: PSS-based OECT (p-OECT) sensors. The bottom electrodes of the OSCs are connected to the source electrodes of the sensors. The platinum gate is changed by a mixture of chitosan and glucose oxidase for glucose sensing, and the Ca^2+^ selective membrane is coated on the PEDOT: PSS channel for Ca^2+^ selective monitoring (Ca^2+^ OECT, c-OECT). When a positive bias is provided, enzymes immobilized on the gate electrode catalyze the electrochemical oxidation of H_2_O_2_ by the platinum electrode. This produces peroxide (H_2_O_2_) from glucose. By transferring electrons to the gate electrode and altering the electrical double layer at the gate/electrolyte interface, the oxidation process lowers the voltage drop at the interface, which raises the potential applied to the active channel and lowers the drain current [76].

An innovative remotely controllable SCL designed to revolutionize healthcare through noninvasive glucose monitoring and targeted drug delivery for diabetic retinopathy treatment is proposed in [24]. This multifunctional marvel comprises five integral components, each contributing to its remarkable functionality: a real-time electrochemical biosensor, an adaptable on-demand flexible drug delivery system (f-DDS), an efficient resonant inductive wireless energy transfer system, a highly integrated circuit (IC)-based microcontroller chip with an adept power management unit (PMU), and a sophisticated remote radio frequency (RF) communication system, as depicted in Figure 3b. The real-time amperometric biosensor’s pivotal role is to detect glucose levels in tears, obviating the need for invasive and uncomfortable blood tests. Meanwhile, the self-regulated pulsatile f-DDS allows for controlled drug release, with remote communication as its modus operandi. The lens’s wireless powering is facilitated by resonant inductive coupling to a copper (Cu) receiver coil, drawing energy from an external power source equipped with a transmitter coil. The device seamlessly communicates with an external controller via RF communication. In our comprehensive evaluation, we explore and elucidate the potential and feasibility of this cutting-edge SCL in the realms of diabetic diagnosis and the therapy of diabetic retinopathy, marking a remarkable leap forward in healthcare innovation.

### 3.3. Fabrication Methods

The science and technology of systems with integrated channels on the microscale (from tens to hundreds of micrometers) allow for the controlled and systematic manipulation of small amounts of fluid flow in specific configurations [77]. The widespread use of microfluidics in current organ-on-a-chip systems, multiphase flow manipulation, chemical synthesis, and bioanalysis makes it especially appealing for contact lens applications. Microscale forces, including fluid surface tension, capillary forces, energy dissipation, and fluid resistance, are the basis for the principles regulating microfluidics in contact lenses [78]. When the eyelids blink, the person’s tears will cover the surface of the contact lens. Then, due to the capillary force, they will enter microchannels during ocular fluid collection. When liquid moves via a small opening, capillary phenomena take place. A crucial parameter in microchannels that gauges the degree of fluid flow resistance is the Reynolds number [25].

SCLs incorporate numerous microelectronic components and microchannels, which are created either directly or indirectly through the utilization of photolithography technology. Photolithography is a manufacturing process that employs light to transfer patterns from a photomask onto the surface of a silicon wafer. The use of photolithography in SCLs encompasses two primary aspects. Firstly, it involves replicating the desired microstructures within the contact lens material. Secondly, it entails the fabrication of microelectronics, including flexible wires, microelectrodes, and other microsensor components integrated into the contact lenses.

When designing SCLs, it is important to consider the peculiarities of microfluidic systems. For instance, the size/shape and flow rate of microchannels can influence the collection of ocular fluid. As it impacts the fluid absorption efficiency of microsensors and could alter the detection accuracy of microsensors, the flow velocity of the ocular fluid is crucial for optimizing the properties of SCLs. Wu et al. used MEMS technology to directly integrate a flow velocity sensor into microchannels to monitor the flow velocity and analyze the microfluidic flow velocity in SCLs. They employed a microchannel wall that served as both a heating element and a velocity sensor to produce a boron-doped polysilicon sheet [79].

Microfluidics stands at the forefront of innovation in the realm of SCLs, ushering in a transformative era in wearable technology and elevating the visual capabilities to new heights [80]. Nestled within the intricate structure of these lenses are minuscule, precisely engineered channels and chambers, enabling the meticulous control and manipulation of fluids [81]. The integration of microfluidic systems into SCLs unlocks a spectrum of functionalities, from administering targeted drug delivery for ocular conditions to dynamically adjusting focal length for individuals grappling with presbyopia. Moreover, it facilitates the real-time monitoring of biomarkers within the tears, revolutionizing health diagnostics [82]. Beyond these impressive features, microfluidics is the linchpin for ensuring user comfort and safety, maintaining a stable tear film and shielding against discomfort or dryness. As we look ahead, smart lenses fortified with microfluidic prowess are on the brink of reshaping our engagement with vision correction and wearable tech, seamlessly blending convenience, health surveillance, and an enriched visual experience [83].

Injection molding is a method of producing SCLs that combines conventional contact lens production methods with the incorporation of smart technology components. The preferred lens substance is manufactured as a liquid or pellet. It might be a hydrogel made of silicone or another appropriate polymer. When the material is heated and poured into the heated mold cavity, which is then sealed shut under intense pressure, the material takes on the shape of the lens mold. In the mold, the substance is allowed to cool and harden. The lens is released from the mold once it has been set and is then subjected to a few post-processing procedures. To get rid of any flaws and guarantee a smooth surface for pleasant usage, the lenses are polished.

**Figure 4:**
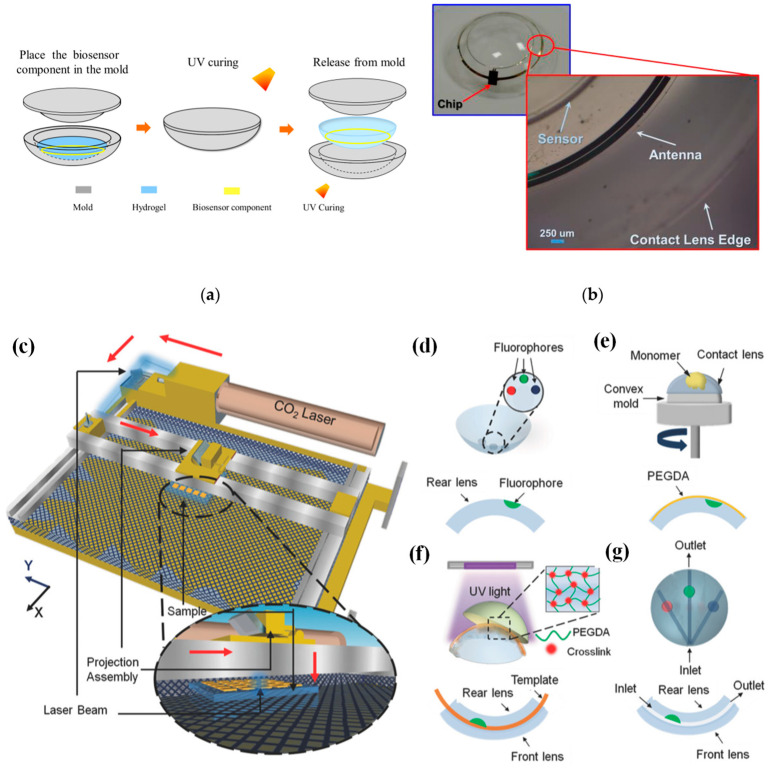
(**a**) Contact lens assembly process [69]. (**b**) Assembled wrinkle-free soft contact lens [69]. The process of fabricating microfluidic contact lenses involves several critical steps: (**c**) to begin, CO_2_ laser patterning is employed, as depicted schematically, with arrows illustrating the laser path [84]; (**d**) subsequently, fluorophores are meticulously deposited within the intricately patterned microconcavities found on the contact lens [84]; (**e**) following this, a PEGDA monomer layer is uniformly applied to the contact lens through a spin-coating process [84]; (**f**) to establish microchannels spanning the microconcavities, fiber templates are strategically positioned, and the combination of these templates with the microconcavities is achieved using UV-initiated free-radical polymerization, ultimately fusing them with a pristine contact lens [84]; (**g**) the final step involves the careful extraction of the fiber templates from the contact lens, resulting in the successful creation of a fully functional microfluidic contact lens [84].

In [69], a conventional cast molding technique was employed to seamlessly integrate the chip, receiving antenna, and biosensor onto a soft contact lens using readily available biocompatible materials. To enhance comfort and user adherence, it was imperative to conform the contact lens design to standard specifications, with thicknesses of 200 μm and 100 μm for the peripheral and optical regions of the lens, respectively. This study utilized a conventional contact lens manufacturing process to create the proposed SCL packaging, employing hydrogel-based materials. Figure 4a illustrates the procedural steps involved in crafting the hydrogel-based contact lens biosensor system packaging, employing a set of convex and concave contact lens molds [69]. During this process, the biosensor component was positioned onto a concave mold, ensuring alignment with the mold’s center. Subsequently, a hydrogel material was poured into the concave mold, and the convex and concave molds were combined. The contact lens, along with the molds, was then subjected to ultraviolet (UV) light for curing. Finally, the mold underwent immersion in a hydration solution to facilitate the demolding process. Throughout the reaction, the contact lenses within the molds absorbed water, rendering them pliable and easily detachable from the molds. This assembly process seamlessly integrated with the standard contact lens manufacturing process, resulting in improved edge smoothness and flatness of the contact lens. This method played a crucial role in achieving a wrinkle-free SCLs, as depicted in Figure 4b [69].

In [84], CO_2_ laser ablation (10.64 µm wavelength, 30 W power) with a spot size of approximately 180 µm was opted to create microconcavities in contact lenses due to its advantages of rapid fabrication and precise programmable beam speed and accuracy (as illustrated in Figure 4c). To craft the microfluidic contact lens, two standard commercial contact lenses were employed. The fabrication process commenced by using laser ablation to create microconcavities on a contact lens, followed by the introduction of fluorophores into these microconcavities, as shown in Figure 4d. Subsequently, a layer of poly(ethylene glycol) diacrylate (PEGDA) monomer was evenly applied through spin-coating onto the contact lens, which was affixed onto a convex mold (as depicted in Figure 4e). Within the PEGDA layer positioned above the patterned microconcavities, silica fiber templates were inserted. These templates were then enclosed between another unaltered contact lens, which served as the front surface of the assembly. The front and rear contact lenses were securely bonded together through a process of UV-initiated free-radical polymerization (depicted in Figure 4f). To complete the creation of the microfluidic device, the silica fiber templates were carefully removed from the contact lens, resulting in the final product, as illustrated in Figure 4g.

Laser ablation is a frequently employed method in the production of microfluidic devices. This technique involves directing a high-intensity laser beam at specific locations on materials, allowing the laser’s energy to remove material at the targeted points. The creation of a microstructure is achieved by adjusting the laser source’s position or projecting the laser through a mask onto the substrate, considering factors such as the substrate material, laser intensity, and wavelength. For the first time, femtosecond laser ablation was harnessed as a straightforward, one-step, and incredibly precise method for crafting NFC antennas using conventional flexible printed circuit board materials [85]. This innovative approach allowed us to create antenna lines with a depth of 9 μm and a width of 35 μm. The resulting antenna, boasting a compact footprint of 19.5 mm^2^, was subjected to rigorous testing in biological solutions, enduring aging, and bending trials. Remarkably, the antenna exhibited a frequency deviation of less than 1%. In a real-world application, the potential of this technology is showcased by fabricating a SCL integrated with the NFC antenna, an NFC chip, and an electrochemical sensor. This SCL enabled the wireless monitoring of glucose levels in an artificial tear solution via a smartphone. Impressively, the device demonstrated its capability to accurately quantify biologically relevant glucose concentrations within the range of 0.2 to 1 mM, with a limit of detection as low as 66 μM. Furthermore, the device exhibited a minimal response to interfering molecules, with a variation of less than ±1 nA, and successfully passed a spike-and-recovery test [85].

Chiou et al. detailed the creation of a graphene-based thin-film supercapacitor designed for use as an energy storage system in a SCL [86]. The fabrication process involved several steps: the initial deposition of copper and parylene-C layers on a silicon wafer as sacrificial layers; the deposition and patterning of Ti (40 nm) and Au (200 nm) to form the current collector; the application, baking, and patterning of graphene; the coating of a PVA-H3PO4 gel electrolyte through drop-casting; the subsequent deposition of parylene-C via chemical vapor deposition (CVD) for electrolyte insulation; the release of the components from the silicon wafer and their incorporation into a standard hydrogel soft contact lens using a cast-molding process. This innovative approach resulted in a SCL characterized by stability and flexibility, making it a potential substitute for RF-based power systems.

Each lens is thoroughly examined for flaws, shape accuracy, and the integration of smart components before being packaged in a sterile setting to preserve its safety and quality until it is worn. It may be necessary to calibrate and test the electronic components of SCLs. This can entail making sure that the sensor readings are correct, the wireless connection is effective, and the power usage is minimized. To provide optimal vision correction and comfort, SCLs must be fitted to the wearer’s eye and prescribed by an eye care specialist. The guidelines for wearing and caring for SCLs must be followed by the wearers. This entails treating them gently, charging them if they have power, and replacing them according to the manufacturer’s instructions.

### 3.4. Applications

Diabetes stands as one of the most prevalent lifelong chronic conditions afflicting humans. Its primary causes are rooted in genetic predisposition, immune system dysregulation, and other factors impacting the human body. These factors collectively contribute to the decline in islet function and the emergence of insulin resistance, culminating in an imbalance of glucose levels within the body. This manifests as a disruption in glucose metabolism and the onset of hyperglycemia. Diabetes encompasses two distinct categories: type 1 and type 2. Type 1 diabetes arises from a deficiency in insulin secretion by the pancreas [87]. Conversely, type 2 diabetes predominantly stems from the ineffective utilization of insulin, driven by insulin resistance and diminished insulin sensitivity in affected individuals. The prevalence of diabetes is substantial, with a wide array of complications and multifaceted underlying causes. It poses significant challenges in terms of treatment and presents considerable threats to human health. As a result, numerous fields of research have been actively engaged in the study and exploration of diabetes-related aspects [88].

**Figure 5:**
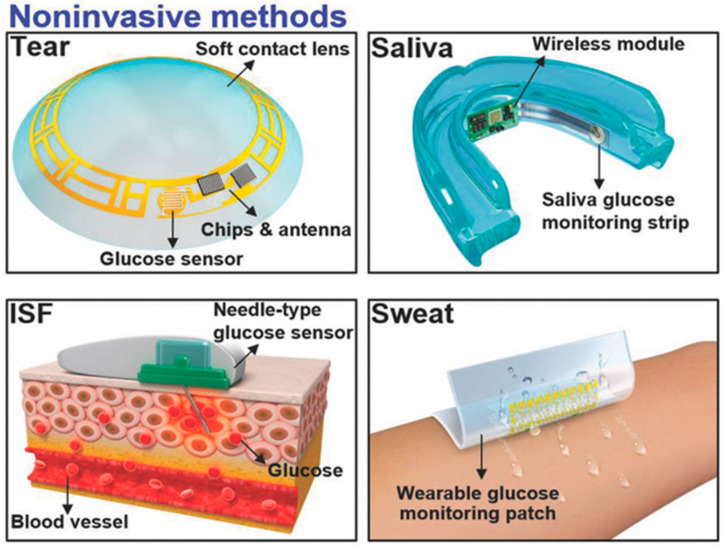
Non-invasive methods are employed by utilizing tears, saliva, ISF, and sweat [95].

The measurement of glucose concentration plays a crucial role in diagnosing diabetes mellitus. Nevertheless, the conventional method of obtaining a single-time-point blood glucose reading necessitates a painful finger puncture to collect a blood sample. Biological fluids, such as tears [89], saliva [90], interstitial fluid (ISF) [91,92], and sweat [93,94], offer non-invasive or minimally invasive methods for obtaining samples, as shown in Figure 5 [95]. These fluids contain glucose concentrations that correlate with blood glucose levels, making them intriguing candidates for painless glucose monitoring within the body. Nevertheless, ensuring the accuracy and reliability of glucose measurements from these alternative biofluids requires careful consideration. One critical aspect to note is that the glucose levels in these biofluids tend to be lower compared to blood.

Continuous glucose monitors (CGMs), often referred to as diabetes monitors, represent wearable technological advancements designed to simplify the continuous tracking of blood sugar levels [96]. These FDA-approved medical devices regularly assess glucose levels in your bloodstream while they are worn. The functioning of a CGM relies on a minuscule sensor that is discreetly inserted beneath the skin, typically on the abdomen or arm. This sensor gauges the interstitial glucose concentration, which refers to the glucose present in the fluid that surrounds cells. Every few minutes, this sensor dutifully records the glucose levels, with the collected data being wirelessly transmitted to a monitoring device. The monitoring device can be conveniently carried in a pocket or purse as a standalone unit or as an integral component of an insulin pump. In some instances, certain CGMs can transmit data directly to a tablet or smartphone [97].

Extensive research and data have consistently demonstrated a positive correlation between the glucose concentration in tears and that in the blood [98]. To mitigate the discomfort of blood sampling, SCLs equipped with an integrated glucose sensor offer an innovative solution. These lenses can continually monitor the glucose concentration in tears, providing a less invasive and more user-friendly alternative for managing diabetes. Within human tears reside a multitude of compounds encompassing proteins, lipids, electrolytes, urea, ascorbic acid, L-lactic acid, cholesterol, and other pivotal metabolites. Remarkably, the chemical makeup of tears closely mirrors that of blood. The real-time assessment of the concentrations of these substances furnishes essential physiological insights, thereby contributing to the enhancement of approaches aimed at treating and preventing certain illnesses. The most vital analytes and their concentration in the tears along with the related diseases are listed in Table 2.

Moreover, research has underscored the significant role of substantial fluctuations in IOP as a contributing factor to the development of glaucoma [111,112]. However, the routine measurement of IOP using the Goldmann applanation tonometer, a conventional clinical tool, is a complex procedure. Consequently, contact lens sensors have emerged as a promising avenue for IOP monitoring by gauging changes in corneal curvature. Leonardi et al. devised a wireless SCL engineered to detect IOP. This innovative lens comprised components such as an antenna, passive gauges, a microprocessor, and other electrical elements. Notably, this device facilitated prolonged and minimally invasive IOP monitoring, delivering both diagnostic and therapeutic advantages for effective glaucoma management. In the realm of IOP monitoring using contact lenses, four fundamental types of sensors have emerged: capacitance sensors [113], piezoresistive sensors [114], strain gauge sensors [115], and micro-inductor sensors [37]. Each of these sensor types holds the potential to contribute to enhanced IOP monitoring techniques.

Lactate stands as a pivotal biomarker with considerable significance in clinical diagnostics and health surveillance [116]. Its utility spans the identification of hypoxia or elevated salt levels arising from physiological or pathological circumstances. Broadly speaking, a human blood lactic acid level surpassing 2 mmol/L is indicative of lactic acidosis, a condition associated with potential complications, such as lactic acid poisoning, stemming from factors like toxins, shock, anemia, sepsis, and organ failure. In the contemporary landscape, tears have emerged as a viable alternative sampling medium due to their ease of extraction, offering a more comfortable and less burdensome substitute for the traditionally uncomfortable blood sampling method [31,99,100]. The realm of real-time lactate concentration monitoring within the body has gained traction through the utilization of contact lens sensors. This avenue holds promise, presenting an exciting prospect for the future.

#### 3.4.1. SCLs for Glaucoma Monitoring and Treatment

**Figure 6:**
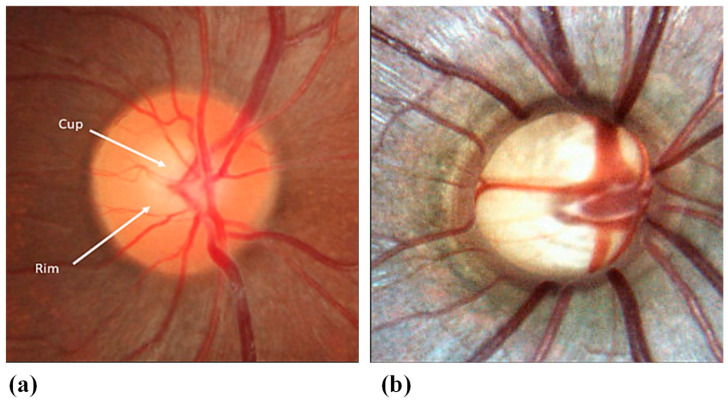
(**a**) Normal optic nerve; (**b**) glaucomatous optic nerve with cupping [118].

Glaucoma encompasses a collection of eye disorders that can result in harm to the optic nerve and loss of vision. The optic nerve is accountable for conveying visual data from the eye to the brain [117]. An image of a healthy optic nerve is shown in Figure 6a [118]. In most glaucoma cases, harm to the optic nerve is associated with heightened pressure inside the eye, known as intraocular pressure. The equilibrium between the production and outflow of aqueous humor is known as the IOP. The ciliary body, a tissue structure behind the iris that also suspends the intraocular lens, produces aqueous humor, the intraocular fluid needed to nourish the eye’s tissues. The anterior chamber is where the secreted aqueous humor enters from behind the iris, travels through the pupil, and exits the eye via the drainage angle.

The optic nerve develops distinctive cupping visible in ophthalmoscopy because of the retinal ganglion cell (RGCs) and RGC axons’ slow deterioration, as shown in Figure 6b [118]. Nonetheless, glaucoma can also manifest with normal or low IOP levels. Glaucoma appears in various forms, but the central categories are open-angle glaucoma and angle-closure glaucoma [119].

Open-angle glaucoma is the most prevalent. It arises when the drainage angle within the eye becomes less effective at permitting the outflow of fluid (aqueous humor) from the eye, leading to a gradual elevation in the IOP. This increased pressure can result in progressive damage to the optic nerve, causing peripheral vision loss that may advance to central vision loss if not treated. Whereas angle-closure glaucoma is less common but can be more abrupt and severe. It emerges when the drainage angle of the eye becomes obstructed or blocked, inducing a rapid surge in the IOP. This can cause symptoms such as intense eye pain, headaches, blurry vision, and nausea. Angle-closure glaucoma necessitates immediate medical intervention as it is a critical condition.

Certain SCLs designed for IOP monitoring possess added attributes, including traits like transparency and stretchability [120,121,122]. Moreover, there are drug-dispensing SCLs that significantly enhance drug effectiveness compared to traditional eye drops. This is achieved by preventing drug loss due to blinking and tears, resulting in increased drug bioavailability [24]. In the context of glaucoma treatment, SCLs incorporating drug complexes [123] and micelles [124] have also emerged. Nonetheless, these innovative lenses encounter certain obstacles that limit their broader application. Such challenges encompass issues like inadequate sensitivity, the necessity for improved biocompatibility, and enhanced stability to enable prolonged and reliable IOP monitoring without the reliance solely on on-demand drug delivery mechanisms.

**Figure 7:**
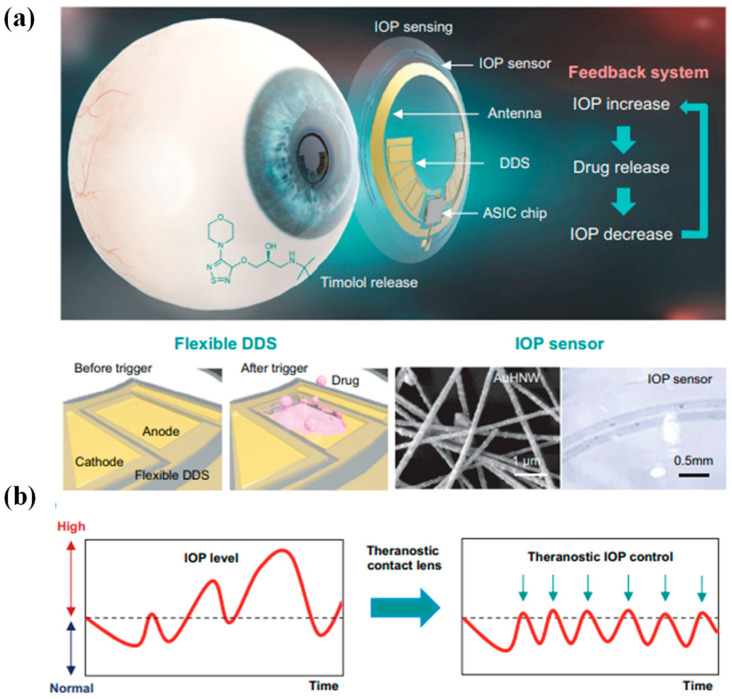
Diagrammatic depiction of a theragnostic SCL devised for addressing glaucoma. (**a**) The design encapsulates a comprehensive arrangement comprising a gold hollow nanowire-based IOP sensor, a drug delivery system (DDS), and wireless circuitry. This amalgamation enables wireless glaucoma treatment, facilitated by a feedback mechanism responsible for IOP assessment and controlled timolol release. (**b**) The schematic delineates a contrast between conventional continuous IOP monitoring and the innovative approach involving IOP regulation through monitoring coupled with personalized drug administration, thereby offering a dynamic strategy for managing glaucoma [36].

Glaucoma stands as one of the irreversible ocular conditions that holds potential for inducing vision loss in severe instances. Despite the commercialization of Triggerfish for monitoring IOP related to glaucoma, the realm lacks an intelligent contact lens capable of overseeing IOP while also facilitating appropriate drug interventions according to pressure fluctuations. This prompts the proposition of an intricately designed theragnostic SCL [36]. This advanced lens encompasses a sensitive IOP sensor built on gold hollow nanowire technology, rendering it highly receptive to ocular strain, as shown in Figure 7a,b. Its chemical stability and biocompatibility further enhance its utility. Complementing this is a flexible drug delivery system, empowering the on-demand administration of timolol to regulate IOP. The lens integrates wireless power and communication systems alongside an application-specific integrated circuit chip. This synergy empowers both the monitoring and control of IOP in glaucoma. Through experimentation on glaucoma-induced rabbits, it has been demonstrated that this innovative theragnostic SCL effectively monitors and manages IOP levels. As a result, this lens emerges as a promising candidate for a forward-looking personalized healthcare framework tailored for addressing glaucoma and other ocular ailments [36].

The continuous monitoring of IOP, especially while asleep, remains a significant challenge in the care of individuals with glaucoma. A new category of intelligent soft contact lenses has been introduced to address this issue [46]. These innovative lenses allow for uninterrupted 24 h monitoring of IOP, even during sleep. What sets these smart soft contact lenses apart is their integration into various commercially available brands of soft contact lenses. Remarkably, this integration does not alter the lenses’ inherent qualities, such as their lens power, biocompatibility, softness, transparency, wettability, oxygen permeability, and suitability for overnight wear.

These smart soft contact lenses have demonstrated the ability to conform seamlessly to diverse corneal curvatures and thicknesses within human eyes. This adaptability enables them to accurately measure absolute IOP while individuals are on the move. A comprehensive series of assessments conducted in vivo using rabbit, dog, and human eyes, spanning from normal conditions to cases of hypertension, has confirmed the exceptional precision in measurements, consistency within individual subjects, and the high level of user comfort provided by these smart soft contact lenses. These capabilities surpass the capabilities of current wearable ocular tonometers [46].

#### 3.4.2. SCLs for Continuous Glucose Monitoring

SCLs have the potential to revolutionize glucose monitoring for individuals with diabetes [22]. The idea is to embed sensors and technology within the lens that can continuously monitor glucose levels in tears, offering a non-invasive and convenient way to track blood sugar levels [125]. Smart lenses equipped with glucose sensors can continuously measure glucose levels in tears, providing real-time data on changes in blood sugar. This continuous monitoring offers a significant advantage over traditional finger-prick tests, which provide only snapshots of glucose levels. Moreover, traditional glucose monitoring methods involve pricking the finger to obtain a blood sample. Smart lenses eliminate the need for such invasive procedures, reducing discomfort and the risk of infections [125,126].

CGM through smart lenses could provide a more comprehensive view of glucose fluctuations throughout the day and night [76]. This information can help individuals make better-informed decisions about their diet, exercise, and medication to achieve better glycemic control. With smart lenses, individuals with diabetes would not need to carry around separate glucose monitoring devices or remember to perform frequent finger-prick tests. The data would be available directly through the lens. SCLs could be programmed to provide alerts when glucose levels reach certain thresholds [127]. This would enable wearers to take immediate action, such as adjusting their insulin dosage or consuming carbohydrates, to manage their glucose levels effectively. However, it is important to note that developing smart lenses for glucose monitoring comes with technical, scientific, and regulatory challenges. Ensuring accuracy and reliability in glucose measurements, addressing potential discomfort or irritation caused by the lens, securing data privacy, obtaining regulatory approvals, and achieving cost-effectiveness are among the challenges that need to be overcome [128].

Despite extensive research into the utilization of SCLs for diagnostic purposes, there has yet to be any documentation regarding the integration of electrically regulated drug administration alongside real-time biometric analysis. A novel SCL has been developed to fulfil both continuous glucose monitoring and the treatment of diabetic retinopathy [24]. Constructed using a biocompatible polymer, this SCL device incorporates ultrathin and flexible electrical circuits, as well as a microcontroller chip, enabling real-time electrochemical biosensing, on-demand controlled drug release, wireless power management, and data communication. Through experimentation on diabetic rabbit models, glucose levels in tears were successfully determined, corroborated by conventional invasive blood glucose tests, and the dispensing of drugs from reservoirs to address diabetic retinopathy. This study effectively validates the feasibility of employing SCLs for non-invasive, continuous diabetic diagnosis, and the therapeutic management of diabetic retinopathy [24].

SCLs are gaining significant interest since they can directly monitor physiological and environmental data [129]. Prior demonstrations, however, frequently lacked simple production processes, reliable mechanical stability, or biocompatibility. However, a flexible method for fabricating multifunctional SCLs is proposed using a serpentine mesh sensor system based on ultrathin MoS_2_ transistors [5]. A photodetector for receiving optical data, a glucose sensor for measuring blood sugar levels directly from tear fluid, and a temperature sensor for spotting potential corneal diseases are all included in integrated sensor systems. This serpentine mesh sensor system, which delivers high detection sensitivity while being mechanically robust and not interfering with either blinking or vision, can be directly mounted onto the lenses and maintain direct contact with tears, in contrast to traditional sensors and circuit chips sandwiched in the lens substrate. Additionally, the in vitro cytotoxicity studies show good biocompatibility, suggesting that these next-generation soft electronics have a bright future in the fields of healthcare and medicine [130].

**Figure 8:**
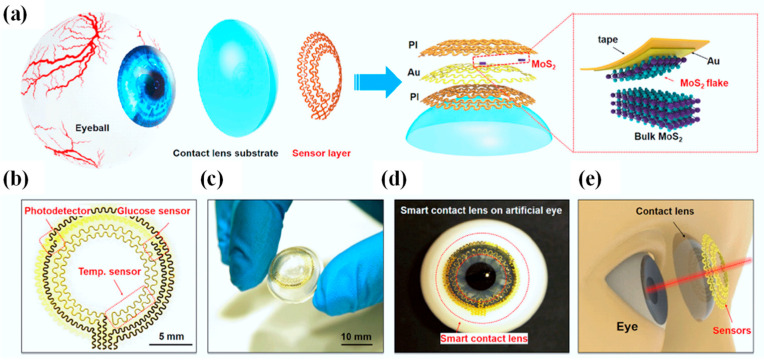
Design and structure of an innovative SCL featuring ultrathin MoS_2_ transistor-based serpentine mesh sensor system [5]. (**a**) Visual representation of the multi-layered configuration of the SCL attached to an eyeball. The highlighted area shows the process of gold-mediated mechanical exfoliation of a monolayer MoS_2_ [5]. (**b**) An optical image displaying the intricate serpentine electrode and sensor arrangement [5]. (**c**) A photograph displaying the sensor layer skillfully transferred onto a curved PDMS substrate [5]. (**d**) An image illustrating the system positioned on an artificial eye [5]. (**e**) An illustrative depiction of the SCL along with its associated dimensions concerning the human eye [5].

Figure 8a presents an overview of the design and structure of the SCL, which consists of a sensor layer with a donut-shaped configuration and a PDMS lens substrate affixed to the eyeball [5]. Within the sensor layer, hybrid sensors employ a serpentine interconnect of gold (Au) electrodes (150 nm), effectively shielded or supported by one or more thin layers of polyimide (PI) (2.5 µm per layer) (refer to Figure 8a, right). This serpentine mesh structure not only offers remarkable flexibility and stretchability but also ensures that the mechanical plane remains neutral, thereby minimizing uniaxial strain when the structure undergoes slight bending. The sensor layer comprises a MoS_2_ photodetector, a MoS_2_ glucose sensor, and an Au temperature sensor, all interconnected through pairs of serpentine metal electrodes (as depicted in Figure 8b).

The accompanying photograph in Figure 8c exhibits the sensor layer positioned atop a dome-shaped PDMS substrate (30 µm), displaying excellent adhesion and bonding to the surface. PDMS, being one of the most widely employed silicon-based organic polymers, has found extensive utility as a substrate across various biomedical domains, including the development of contact lenses. In comparison to alternative commercial materials employed in contact lens manufacturing, such as polymethyl methacrylate, polyvinyl acetate, rigid gas-permeable materials, and hydrogels, PDMS stands out due to its cost-effectiveness, ease of production, durability, comfort when worn, and high oxygen permeability. Its primary drawback, limited hydrophilicity, can be ameliorated through methods like plasma treatment, grafting of hydrophilic polymers, or surface modification incorporating embedded surfactants. Figure 8d captures an image of the intelligent lens placed on an artificial eye, clearly indicating that the sensors do not obstruct the pupil, thereby ensuring unobstructed vision (as illustrated in Figure 8e) [5].

SCLs designed for medical and personal use require small power sources. Integrated batteries offer promising options for these devices. However, charging batteries in small wearables presents challenges due to the difficulty of transferring electrical power through miniaturized wired connections or wireless transmission units. A proposed solution involves using safe tear-based batteries that are integrated into the contact lenses and charged by biofuel during storage [128]. To charge the cathode and anode, the battery utilizes enzymatic reactions of glucose oxidase and self-reduction of conducting polymer, respectively. The contact lens contains embedded electrodes that are discharged in an artificial tear solution and then charged in a glucose solution through a bio-reaction process known as bio-charging. The bio-charging battery demonstrates a discharging capacity of 45 μA cm^−2^ and a maximum power of 201 μW cm^−2^. Its performance has been verified over 15 cycles. Additionally, the bio-chargeable battery can also be charged conventionally using an external power supply [128].

Previous research studies have primarily focused on designs that incorporated electronic devices or antennas for wireless transmission. These designs are power-intensive and require external receivers around the ocular system. To address these limitations, a power-free SCL has been developed for non-invasive glucose sensing [127]. This innovative lens utilizes multiple electrochromic electrodes to detect glucose levels and transmit data without the need for an external wireless system. The device can detect a wide range of glucose concentrations, from normal levels (0.16–0.5 mM) to abnormally high concentrations (0.9 mm). The multi-electrode design demonstrates excellent accuracy, with a correlation coefficient of 0.99543 when compared to controlled samples. It also enables the detection of low glucose concentrations as low as 0.05 mm. Moreover, the device exhibits good reproducibility, with standard deviations of determined glucose levels measuring 0.0462 and 0.025 for four continuous cycles and extended intervals of several days, respectively. This breakthrough SCL has the potential to revolutionize daily health monitoring for ordinary users. It eliminates the need for a power supply and external devices, making it a convenient and accessible option for widespread use. Furthermore, its simple electronics-free structure allows for cost-effective manufacturing, paving the way for its immediate application in the market [127].

Recent breakthroughs in wearable electronics, in tandem with wireless communication technologies, play a pivotal role in realizing medical applications through health monitoring innovations. For instance, envision a SCL capable of continuously monitoring eye physiology and tear fluid, offering real-time, noninvasive medical diagnostics. However, prior reports on SCLs have noted the use of opaque and brittle components to facilitate electronic functionality, potentially obstructing vision and posing risks to eye health. Furthermore, the reliance on costly, bulky equipment for sensor data retrieval can disrupt users’ daily activities.

**Figure 9:**
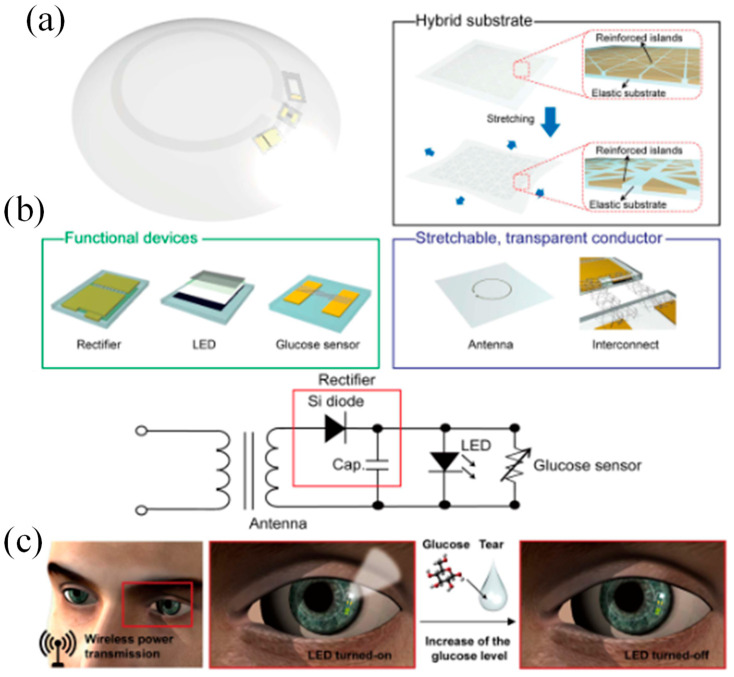
Illustrates the stretchable and transparent SCL system. (**a**) The soft, SCL is depicted in a schematic illustration. It comprises a hybrid substrate, functional devices such as a rectifier, LED, and glucose sensor, as well as a transparent and stretchable conductor used for antennas and interconnections [31]. (**b**) Circuit diagram of the SCL system is presented [31]. (**c**) The operation of this soft SCL is explained. Electric power is wirelessly transmitted to the lens via the antenna. This power activation, in turn, activates the LED pixel and the glucose sensor. Upon detecting a glucose level in tear fluid above a certain threshold, the pixel is designed to turn off [31].

In response, an unconventional approach is proposed for crafting a pliable SCL [31]. This innovative design seamlessly integrates glucose sensors, wireless power transmission circuits, and real-time sensing signal visualization via transparent and flexible nanostructures. The incorporation of this display directly into the smart lens eliminates the need for additional cumbersome measurement devices. Consequently, this soft, intelligent contact lens maintains transparency, ensuring a clear view by aligning the refractive indices of its locally patterned regions. The resulting soft, SCL boasts real-time wireless functionality, as confirmed by in vivo tests for monitoring glucose concentrations in tears, particularly suitable for assessing fasting glucose levels in diabetic patients. Simultaneously, it offers sensing results via the contact lens display, revolutionizing the realm of health monitoring. Figure 9a provides an overview of the soft, SCL layout, showcasing the seamless integration of a glucose sensor, wireless power transfer circuit, and display pixel through the use of transparent and stretchable interconnections. The central concept underlying this soft, SCL system is its wireless health monitoring capability, specifically in monitoring the glucose levels in the wearer’s tears, which is achieved through the LED pixel.

Figure 9b presents the circuit diagram of the SCL. In this advanced lens, the antenna receives radio frequency (RF) signals from a transmitter with a transmission distance of less than 9 mm. The rectifier, which is a combination of a diode and a capacitor, converts the alternating current (AC) signals into direct current (DC) to activate the LED and operate the sensor. The primary mechanism for detecting glucose levels in this contact lens is depicted in Figure 9c. When exposed to tear fluid with a glucose concentration exceeding a certain threshold, the sensor’s resistance decreases. This reduction in resistance results in a decrease in the parallel circuit’s resistance, which includes the LED and sensor, while the resistance of the other components in the system (antenna and rectifier) remains constant. This difference in bias applied to the LED pixel under a constant voltage condition determines whether it is turned on or off. Table 3 provides several important works on SCLs for glucose and glaucoma monitoring and treatment. 

## 4. Current Challenges and Prospects

A viable platform for non-invasive point-of-care diagnostics has also been developed using nanotechnology in contact lenses. A recent research topic in bioengineering and cutting-edge medical procedures involves using nano-based contact lenses for ocular medication delivery [56]. Despite all the research that has been carried out in this field, new technologies are still in the early phases of development, and additional clinical trials must be conducted before commercializing contact lenses based on nanotechnology [135]. We are persuaded that these lenses embody both technical and material innovations that pave the way for the next generation of precision medicine-oriented devices [136].

SCLs offer numerous advantages, but they are accompanied by certain drawbacks. These lenses tend to be notably pricier than traditional lenses, encompassing not only the lenses themselves but also associated devices and maintenance. SCLs are more intricate compared to standard ones, making them potentially more challenging to use and maintain. Users may need to acquire knowledge on how to operate them, troubleshoot technical issues, and ensure they remain clean and charged. Many SCLs require a power source, typically a small battery. Battery life may be limited, particularly when supporting features like augmented reality displays or continuous health monitoring. Recharging or replacing these batteries can be inconvenient.

The integration of technology into contact lenses can impact their comfort and fitness. Some users may experience discomfort or eye irritation with SCLs. If not used and cared for correctly, SCLs can pose health risks like infections or corneal damage. Extended wear or inadequate hygiene can exacerbate these risks [137]. Devices like SCLs have the potential to collect sensitive user data, including biometric information and audiovisual recordings. Concerns may arise regarding who can access this data and how it is utilized. Moreover, not all SCLs may be compatible with every device or operating system, limiting their usability for certain users. SCLs, particularly those with health-related features, may face regulatory hurdles and approval processes, potentially delaying their market entry and increasing costs. The small size and intricate design of SCLs may render them less accessible or functional for individuals with specific physical disabilities [135].

One of the companies actively engaged in the development of smart contact lenses has made a significant strategic decision. Mojo Vision, in a recent announcement, revealed its intention to “realign its business and channel its resources” towards the MicroLED display technology it had pioneered during the course of its work on the Mojo Lens (as reported by Axios). Regrettably, this strategic shift entails the regrettable necessity of downsizing its workforce by approximately 75 percent, as detailed in a communication from the company’s CEO, Drew Perkins [138].

The communication elucidates that the impetus behind this change stems from Mojo’s inability to secure additional funding for the continued development of its smart contact lens venture. Perkins attributes this challenge to a combination of factors, including the sluggish state of the global economy, exceedingly stringent capital markets, and the yet unproven market potential for advanced augmented reality (AR) products. These circumstances have collectively rendered Mojo Vision unable to secure additional private investments required to advance their smart contact lens project.

When Mojo Vision showcased the Mojo Lens at CES 2020, it was evidently still in the developmental stage, some distance away from reaching the consumer market. The demonstration unit, which boasted an impressive 14,000 pixels per inch screen, performed its intended functions effectively. However, a significant drawback was that it necessitated connections to an external power source and processor. This limitation was far from ideal for individuals seeking a practical and wearable solution [138].

Fast forward to June 2022, Mojo achieved a notable milestone by unveiling a “feature-complete prototype” worn by CEO Perkins [139]. This version boasted onboard power and communication capabilities, marking a significant step forward in the development process. However, in a March blog post, the company outlined its ongoing journey, which includes extensive user testing, software application prototyping, and comprehensive system and product optimization. This implies that it will still be some time before consumers can expect to purchase these lenses, suggesting that the product is not yet ready for mass-market availability. Even if Mojo had managed to launch its SCLs, it would not have been a guaranteed success. The concept of wearing face-mounted computers, despite the allure of features like informational overlays, directions, and zooming in on objects, remains uncertain in terms of public acceptance. The memory of Google Glass, which was largely rejected by the general public, still lingers. Furthermore, even if the notion of such technology is now more palatable, Mojo would have faced formidable competition. Meta, for instance, is making substantial investments in AR and actively acquiring companies within the smart glasses sector. While smart glasses may not evoke the same futuristic appeal as SCLs, they can theoretically deliver similar functionality in a slightly different form factor.

Moreover, Mojo is not the sole contender in the MicroLED display market. Industry giants like LG and Samsung have already announced MicroLED TVs, although these displays are considerably larger than what Mojo has showcased. For example, Samsung presented its 76-inch MicroLED CX as the “world’s smallest and most affordable MicroLED screen” at CES this year. The smallest MicroLED TV they exhibited was 50 inches, still substantially larger than something that could fit on your eye. These TVs are typically high-end and come with price tags well above USD 120,000.

Mojo’s pivot away from SCLs is not an isolated case. Verily, a subsidiary of Google’s parent company Alphabet, halted its research on contact lenses designed to monitor wearers’ glucose levels back in 2018, underscoring the challenges inherent in developing eye-based wearables [138]. In collaboration with pharmaceutical giant Novartis, Verily embarked on the development of this lens in 2014 [140]. In a recent blog post, Verily revealed that their measurements of glucose levels in tears, when compared to blood glucose levels, did not meet the required proximity. This innovative lens was comprised of a miniature wireless chip and glucose sensor, nestled between two layers of lens material. Verily explained, “The clinical trials for the glucose-sensing lens showed that our measurements of tear glucose and blood glucose concentrations lacked the necessary consistency to meet the standards of a medical device.” While Verily encountered several challenges in obtaining dependable readings from the eye, they expressed their commitment to exploring alternative applications for the smart lens technology. One such application includes a lens designed to enhance vision post-cataract surgery [140].

Overcoming the multifaceted challenges associated with the development of SCLs demands a comprehensive and innovative approach. Firstly, addressing the technical hurdles involves refining the microfabrication processes to create intricate microfluidic structures and integrating them seamlessly into the lenses, ensuring comfort and durability [15]. Secondly, power sources and energy-efficient components must be developed to sustain the continuous operation of sensors and displays without burdening the wearer [20]. Additionally, data security and privacy concerns require robust encryption and authentication protocols to safeguard sensitive information transmitted by these lenses [141]. Regulatory and ethical considerations also necessitate close collaboration with healthcare authorities to establish standards and guidelines for smart lens technologies. Finally, fostering public trust and acceptance necessitates transparency in terms of data usage and potential health implications. By combining advances in materials, electronics, regulation, and communication, we can pave the way for the successful development and widespread adoption of SCLs, unlocking their immense potential for healthcare, AR, and beyond.

## 5. Concluding Remarks

The eye is a sophisticated organ of the body that contains a variety of metabolite indicators, like glucose, peptides, and specialized ions, as well as numerous vital biological data points, like intraocular pressure, corneal temperature, and pH. The development of SCLs was made possible by considerable breakthroughs in the biocompatibility of material, better lens designs, the healthcare system, and more adaptable and effective modalities throughout the past three decades of contact lens research and patient care. SCLs are sophisticated visual prostheses that can be adjusted and track a variety of significant physical and biochemical alterations in ocular illnesses over a continuous and non-invasive period. The development of wearable technologies that use bodily fluids like sweat, tears, saliva, and electrochemical interactions to continuously monitor physiological conditions and sickness is now underway. Tear fluid is frequently used to evaluate eye problems, blood sugar levels, and even cancers because of its simplicity of access, manufacture, and non-invasiveness. Contact lenses, typically worn to address vision correction and aesthetic preferences, have evolved to serve an additional distinctive role as wearable devices for the real-time monitoring of glucose levels in individuals with diabetes. Beyond the physical composition of these contact lenses, their capacity to function as point-of-care, portable devices hold significant importance in the context of blood glucose monitoring. Key considerations encompass their portability, affordability, and user-friendliness, facilitating swift and precise diagnoses to diminish both time and expenses incurred. This review delves into the extensively explored applications of SCLs, providing a comprehensive discussion of their usage. In addition to highlighting their various applications, we also address the current challenges and prospects within the field.

## Figures and Tables

**Table 1:** Properties of commonly used contact lens materials [44].

Material	Molecular Formula	Procs/Cons	References
PMMA	(C_5_H_8_O_2_)_n_	Outstanding optical properties, low oxygen permeability, high rigidity and toughness.	[61]
PET	(C_10_H_8_O_4_)_n_	Low glass transition temperature, low rigidity, low surface energy, hydrophobic, excellent chemical resistance, and thermal stability.	[62]
PHEMA	(C_6_H_10_O_3_)_n_	Tunable mechanical properties, relatively high water content, and good chemical and thermal stability.	[55]
PDMS	(C_2_H_6_OSi)_n_	Flexibility and high oxygen permeability.	[63]
2-methacryloyloxyethyl phosphorylcholine (MPC)	C_11_H_22_NO_6_P	Low protein adsorption, good surface wettability, high oxygen permeability, and mechanical weakness.	[64]
Chitosan	(C_6_H_11_NO_4_)_n_	Bioadhesive, biocompatible, biodegradable.	[65]

**Table 2:** The concentration of analytes in tears and their related illnesses. Inspired by [22].

Analytes	Tear Conc. (mM)	Diagnostic Disease	References
Lactate	2.0–0.05	Cancer; sepsis; ischemia; liver disease	[99,100]
Glucose	0.01–0.05	Diabetes	[101,102]
Urea	3.0–6.0	Renal function	[103]
Dopamine	0.37	Glaucoma	[36,104]
Cortisol	1–40 ng/mL	Stress levels and brain injuries	
Mg^2+^	0.5–0.9	Hyper/hypomagnesemia	[105]
K^+^	20–42	Hyper/hypokalemia and an indicator of ocular disease	[106]
Ca^2+^	0.4–1.1	Hyper/hypocalcemia	[107]
Cl^−^	118–135	Hyper/hypochloremia	[108]
Na^+^	120–165	Hypo/hypernatremia	[109]
Total protein	7 g/L	Dry eye syndrome	[110]

**Table 3:** Recently proposed SCLs for glucose and glaucoma monitoring and treatment.

Device	Material	Power Source/Sense Type	Application	Functionality	References
SCL	Bimetallic HA-Au@Pt electrode	Wireless	Diabetes diagnosis	Single	[131]
SCL	CuHCFe and GOx	Biofuel	Diabetes diagnosis	Single	[128]
SCL	Multiple electrochromic electrodes	Power-free	Glucose sensing	Single	[127]
SCL	AgNW channel	Piezoresistive strain	Intraocular pressure monitoring	Single	[115]
SCL	PEDOT:PSS	Organic solar cells	Glucose and calcium ions	Single	[76]
SCL	Hydrogel	Battery-free	Intraocular pressure monitoring	Single	[37]
SCL	Anodic aluminum oxide	Power-free	Glaucoma diagnostic and drug delivery	Multi	[132]
Optical fiber sensor integrated in SCL	-	-	Intraocular pressure measurement	Single	[133]
Photonic crystal-based SCL	PC-embedded PDMS membrane	RF-based wireless power/visual color change	Intraocular pressure monitoring	Single	[134]
SCL	PHEMA hydrogel, Gox, BSA, PVA, chitosan	Wireless	Glucose monitoring and therapy	Multi	[24]
SCL	Antiopal structure for IOP monitoring; peptide-functionalized AuNBs SERS substrate for MMP-9 detection	Color change	Intraocular pressure and matrix metalloproteinase-9	Multi	[47]

## Data Availability

Not applicable.
